# Central Pain and Complex Motoric Symptoms after Gosarelin Therapy of Prostate Cancer

**DOI:** 10.1100/tsw.2004.197

**Published:** 2004-11-18

**Authors:** G. Ernst, A. Gericke, P. Berg

**Affiliations:** ^1^ Blefjell sykehus Kongsberg, Anestesi, Drammensvei 4, 3612 Kongsberg, Norway; ^2^ Telegrafen Legekontor Myntgt 4 3612 Kongsberg, Norway

**Keywords:** gosarelin, allodynia, central pain, Zoladex™, movement disorder

## Abstract

A 76-year-old man with prostate cancer T3N0M0 and increasing PSA was treated with goserelin three times in a half year. As soon as the first treatment, he described subjective muscle weakness. After the third treatment, he developed complex motoric symptoms and atypical central pain with a likely association to goserelin. His left arm had signs of spastic movement; pain deteriorated after relaxation. The right hand showed muscle cramps under passive movements of the left arm that were not typical for rigor. He felt aching and partial burning pain in his whole body. There were few allodynic areas, mainly in the left arm. Several treatment approaches failed and the patient died some weeks after the first contact with our pain clinic due to pneumonia.

## INTRODUCTION

Central pain and movement disorder syndromes are rarely diagnosed in cancer patients without cerebral tumors or metastases. Such syndromes are not described after hormone treatment. We present a patient with such syndromes after treatment with gosarelin, a GnRH-analog for prostate cancer.

## CASE

A 76-year-old man presented to our clinic with pain problems in 2003. He was in good health until urination problems began in 1996. A prostate cancer (T3N0M0) was diagnosed and a radical open prostatectomy, followed by radiation, was carried out. In addition, he had a rectal tumor, which was checked with a colonoscopy and was histologically an adenoma. All other examinations showed no sign of metastasis. After radiotherapy, he had transient problems with urination.

In 1997, he had increasing values of prostate-specific antigene (PSA), therefore, a treatment with goserelin (Zoladex™) was started. He got the first injection of 10.8 mg March 1997, followed by one injection June 1997 and one August 1997. After the first injection, the patient felt "strange". After the second injection, he got paresthesias on the ulnar side of the left hand. The third injection was followed by an immediate subjective loss of muscle strength in the whole body. An examination after the second injection showed no pathologic changes except subjective symptoms of paresthesia and muscle weakness in the left hand. An X-ray examination of the neck showed unspecific signs of osteochondrosis C3/4.

After the last injection, he experienced increasing muscle weakness first in the left hand, later in the left arm in combination with pain, cramps, and increased muscle tension in his left neck. He had coordination problems and was unable to accomplish simple tasks with the left hand, such as taking off clothes. He had some paresthesias on the ulnar side of the right hand as well. Neurologic examinations showed first few objective signs, later muscle atrophy and joint stiffness. At that moment, cerebral metastasis was discussed. A computer tomogram of the head showed a large 1.5-cm area in the cerebellum with signs of metastasis. A following MR-examination showed larger ventricles that were interpreted as not pathological for his age, some unspecific high signals around the side ventricles with no significance, but no suspect area in the cerebellum.

In the next year, pain and movement problems increased. Physiotherapy helped gradually, but without a long-term effect. In 2000, he was transferred to the neurologic department of the national hospital where a dystonia of the left arm was diagnosed, later the diagnosis was changed to a Parkinson plus–syndrome. Treatment with L-Dopa and Karbogelin was introduced with small effect. Baclofen had no effect. To mobilize the arm, the patient was treated with a plexus catheter which took away some, but not all, pain. Botulinom-Toxin injections had no special effect. A local treatment of injections with NaCl 0.9% had some symptomatic effects. A cryotherapy was tried two times without any longer effect. The pain was partially opioid-resistant. The rigor in the lower extremities, combined with dystonia and spastic cramps in the left arm, increased. The patient, formerly in good health, was completely invalidized, without any cognitive impairment. The responsible medical supervision board of the state interpreted the association between goserelin and the symptoms as likely.

As the patient presented himself at our pain clinic, he was under a current medication of Amitryptilin (20 mg), L-Dopa, Cabagelin, Omeprazol, and Acetominophen. He was cognitively not influenced, but showed a certain latency both on verbal and motorical reactions. The left hand and arm showed pronounced muscle atrophy and joint stiffness. The left arm had signs of spastic movement; pain deteriorated after relaxation. The right hand showed muscle cramps under passive movements of the left arm that were not typical for rigor. He had a ptosis and a hanging mouth corner on the right side that was first described some months ago in combination with an episode, which was described as syncope. His left leg was spastic, his right leg was more relaxed. He showed a rough intention tremor. The pain he described was in the whole body and was aching and partially burning. There were a few allodynic areas, mainly in the left arm. He showed some disarthry. Shortly after the first contact and before other diagnostic tests were performed, the patient got pneumonia and died.

## DISCUSSION

### Central Pain

Central pain is usually defined as a painful syndrome caused by a lesion or dysfunction in the central nervous system[1]. Etiologies include vascular lesion in the brain and spinal cord (infarct, hemorrhage, vascular malformation), multiple sclerosis (MS) or inflammatory diseases other than MS, traumatic spinal cord injury or traumatic brain injury, syringomyelia and syringobulbia, tumors (seldom), abscesses, epilepsy, or Parkinson’s disease (PD)[2]. Epidemiological data show a prevalence of up to 30% after spinal cord injuries and MS, whereas central pain after stroke has a prevalence of 8% and central pain at PD has a prevalence of less than 5%. At quantitative sensory testing (QST), all patients have abnormal sensibility to pain and temperature, but normal thresholds to touch, vibration, and kinesthesia. Pain qualities that are described by patients include burning, aching, lancinating, pricking, cutting, sore, icy feeling, and many others. Pain may be affected or exaggerated by external and internal events (sensory, visceral, emotions) with prolonged after effects. Possible increase of pain can happen after fear, joy, noise, or bright light. Central pain is normally not associated with cancer that is not situated in the nervous system. It also not associated with other paraneoplastic symptoms[3].

Clinical diagnostic criteria for idiopathic PD include asymmetric resting tremor, asymmetric rigidity and asymmetric bradykinesia. The diagnosis is possible at one of the criteria, probable with two, and definite with two of the three symptoms and response to anti-Parkinson drugs[4]. “Parkinson plus” is a group of sporadic degenerative disorders associated with Parkinsonism that are poorly sensitive to L-Dopa. A combination of Parkinsonism and dystonia has been linked to “Parkinson plus”[5].

Pain is a usual feature of PD. Up to 40% of patients with PD have pain and in a minority of individuals, it becomes severe enough to overshadow the motor symptoms of the disorder. Five categories of pain are described: musculoskeletal pain, neuritic or radicular pain, dystonia-associated pain, primary or central pain, and akathitic discomfort[6]. There is substantial discussion if central pain is generally associated with PD. Most reports are anecdotical. In 1985, Schott presented three patients with PD and severe pain, which had “some features of central pain”[7]. PD is not associated with a classical lesion of the central nervous system, but with a dysfunction. Most pains in PD are due to motorical changes and muscle cramps, however, some pains cannot be explained on this basis and seem likely to be neuropathic in nature, such as the reports of oral/genital pain. A small proportion of patients with PD have sensory symptoms (including pain) that precede any clinically apparent motor effects of the disease. Burning pain is often, though not invariably, related to L-Dopa therapy[8]. The classical explanation for the pathogenesis of PD describes a dopamine deficiency in the substantia nigra. An interesting study showed some evidence that both PD and pains with a possibly central component such as trigeminal neuralgia or phantom limb pain can be triggered or caused by thalamocortical dysrythmias[9]. Such dysrythmias can also be interpreted as thalamic dysfunction, which might be an explanation for central pain states without evidence of lesions in the central nervous system.

In our patient, a diagnosis of Parkinson plus was made by specialists of the national hospital. The patient did not have the classical combination of symptoms, which makes the diagnosis rather weak. Another possible diagnosis could be a corticobasal degeneration that normally impresses with a combination of hypokinetic Parkinson syndrome, dysarthry, problems with swallowing, apraxy, focal reflex myoclonies, dystonias of arms and legs, and a cortical sensoric deafferentiation which continues until the patient does not recognize his own hand (“alien hand”)[10]. However, disarthry was discrete and swallow problems absent in this patient. Also, cognitive alterations were clinically absent making this diagnosis rather unlikely. There were no focal pathological changes in MR-exams that could explain the movement disorder the patient developed. With the described feature of pain, allodynia, poor response on opioids, a description of burning and aching pain in the whole body, it fits to a diagnosis of central pain of unknown origin, together with a unspecified movement disorder syndrome that combines some features of Parkinsonism and dystonia.

## Zoladex™ (Goserelin Acetate) as Cause?

Goserelin is a gonadotropin releasing hormone (GnRH) agonist that is used with the meaning of a pharmacological castration. Goserelin is a synthetic decapeptide analog of luteinizing hormone-releasing hormone (LHRH). For experimental purposes, it has been administered subcutaneously as an aqueous solution, but for therapeutic use it is formulated as subcutaneous depots releasing goserelin over periods of 1 (3.6 mg) or 3 (10.8 mg) months. There is no clinically relevant accumulation of goserelin during multiple administration of these depots. It is unnecessary to adjust the dose or administration interval when the depot formulations are administered to elderly patients or to those with impaired renal or hepatic function. Administration of a goserelin 3.6 mg or 10.8 mg depot results in an initial increase of luteinizing hormone (LH) levels and in increases of serum testosterone or estradiol levels in males and females, respectively[11]. This is followed by a decrease in serum LH levels and suppression of testosterone or estradiol to within the castration or menopausal range, respectively. Subsequently, throughout treatment with goserelin depots, serum testosterone or estradiol levels remain suppressed[12]. Goserelin has a good effect on hormone concentrations, PSA, and prognosis of prostate cancer[13]. There is no difference between patients with goserelin or surgical castration[14] or minimal difference (72 vs. 76 on a 100-point quality-of-life scale)[15].

Most reports about adverse effects come from phase II and phase III studies. Especially more symptomatic adverse effects are possibly underestimated in the published studies[16]. Goserelin can have more adverse effects than Tamoxifen and the level of adverse effects and disability can be similar than at cytotherapeutic treatment (CMF)[17]. Typical adverse effects are osteoporotic changes[18], (partially big, [19]), insulinresistance[20], hot flushes, reduction of libido, lethargy[21], memory disturbances (in psychological tests, [22]), severe fatigue (28% of patients in difference to 18% before therapy, [23]), and depression[24]. A typical adverse effect is transient bone pain after onset of treatment. In an open study, 2 of 7 patients got increased bone pain after the first dose[25]. In an open uncontrolled study of 191 patients with T3 or T4-Tumor, 4 patients (2%) got transient bone pain at therapy onset[26]. Similar transient pain was reported by 5 of 95 patients, RCT[27]. There is some speculation if hot flushes are caused by a functional CGRP after treatments[28]. In preclinical studies were low serum concentrations of Mg and Ca in combination with cisplatin[29]. But, in several studies, goserelin seemed in fact to augment quality of life[30]. There exists no other report about central pain or new diagnosed movement disorders after introducing goserelin therapy despite the use of the medicament for several conditions including prostate cancer, chronic pelvic pain, and several gynecological tumors.

Is there a possibility that the goserelin treatment caused onset of central pain in combination with the described movement disorder? The patient, before the onset of treatment, was in good health without any psychosomatic or psychiatric problems and was able to describe three times subjective changes in muscle function. No other reason has been identified. Of course it is possible to develop a usual PD, but his syndrome was quite atypical. We feel that an association between goserelin treatment and the beginning of his neurologic problems is likely.

It is possible that reduced testosterone has an influence on symptoms of PD. Many of the symptoms of testosterone deficiency are nonspecific and overlap with the nonmotor symptoms of PD, such as decreased enjoyment of life, lack of energy, sexual dysfunction, and depression. In an open-labeled pilot study, it was possible to improve deficiency symptoms of PD by testosterone replacement[31]. It is unclear whether testosterone deficiency is comorbid in PD or whether it plays a role in the pathogenesis of disease[32], therefore, it is also unclear if an iatrogenic-induced testosterone deficiency can induce a former subclinical PD. There is some preclinical evidence that estrogens modulate the activity of dopamine in the extrapyramidal system and PD can worsen with estrogen therapy[33], but there is no evidence that PD can worsen at estrogene-depleting therapy. This pathophysiological explanation seems to be speculative, but there is no other good explanation.

## CONCLUSION

The association between central pain, movement disorder, and use of goserelin in this patient seems to be likely. Patients should be monitored for such adverse effects.
